# Distribution of Phenolic Contents, Antidiabetic Potentials, Antihypertensive Properties, and Antioxidative Effects of Soursop (*Annona muricata* L.) Fruit Parts* In Vitro*

**DOI:** 10.1155/2015/347673

**Published:** 2015-12-15

**Authors:** Stephen A. Adefegha, Sunday I. Oyeleye, Ganiyu Oboh

**Affiliations:** Functional Foods and Nutraceutical Unit, Department of Biochemistry, Federal University of Technology, PMB 704, Akure 340001, Nigeria

## Abstract

Soursop fruit has been used in folklore for the management of type-2 diabetes and hypertension with limited information on the scientific backing. This study investigated the effects of aqueous extracts (1 : 100 w/v) of Soursop fruit part (pericarp, pulp, and seed) on key enzymes linked to type-2 diabetes (*α*-amylase and *α*-glucosidase) and hypertension [angiotensin-I converting enzyme (ACE)]. Radicals scavenging and Fe^2+^ chelation abilities and reducing property as well as phenolic contents of the extracts were also determined. Our data revealed that the extracts inhibited *α*-amylase and *α*-glucosidase and ACE activities dose-dependently. The effective concentration of the extract causing 50% antioxidant activity (EC_50_) revealed that pericarp extract had the highest *α*-amylase (0.46 mg/mL), *α*-glucosidase (0.37 mg/mL), and ACE (0.03 mg/mL) inhibitory activities while the seed extract had the least [*α*-amylase (0.76 mg/mL); *α*-glucosidase (0.73 mg/mL); and ACE (0.20 mg/mL)]. Furthermore, the extracts scavenged radicals, reduced Fe^3+^ to Fe^2+^, and chelated Fe^2+^. The phenolic contents in the extracts ranged from 85.65 to 560.21 mg/100 g. The enzymes inhibitory and antioxidants potentials of the extracts could be attributed to their phenolic distributions which could be among the scientific basis for their use in the management of diabetes and hypertension. However, the pericarp appeared to be most promising.

## 1. Introduction

About 382 million people were estimated to be living with diabetes mellitus (DM) globally, with an alarming projection of 471 million people with the disease by the year 2035 [1]. Type-2 diabetes mellitus accounts for over ninety percent of all cases of diabetes mellitus (DM), in both developed and developing countries. It is characterized by hyperglycemia resulting from defects in insulin secretion, insulin insensitivity, or both [2]. Decreasing hyperglycemia represents a major therapeutic approach towards the management of type-2 diabetes. However, the inhibition of key enzymes (*α*-amylase and *α*-glucosidase) involved in the hydrolysis of starch and absorption of glucose may confer nutraceutical solution towards the management of the diseases. Some drugs presently used (acarbose and miglitol) reduce blood glucose level but with accompanied severe pharmacological side effects [3, 4].

One of the long term complications of diabetes is hypertension [5]. Angiotensin-I converting enzyme (ACE) is a zinc metallopeptidase that converts angiotensin-I to angiotensin-II, a potent vasoconstrictor, and breaks down bradykinin, a vasodilator [6]. Inhibition of ACE is considered a useful therapeutic approach in the management of hypertension in both diabetic and nondiabetic patients [6]. Furthermore, ACE inhibitors have been reported to reduce the risk of type-2 diabetes in nondiabetic patients at baseline; bradykinin enhances the responsiveness of both muscle fiber and adipocytes to insulin [6].

Soursop (*Annona muricata *L.) otherwise known as graviola or guanabana is an edible tropical fruit tree that is widely cultivated across regions of the world [7]. Soursop fruit is specially known for its commercial value in the production of juice, candy, and sherbets [8]. The roots of these species are used in traditional medicine due to their antiparasitic and pesticidal properties. Intensive chemical investigations of the leaves and seeds of this plant have resulted in the isolation of a great number of bioactive compounds which were found to display interesting biological including antitumor, cytotoxicity, and antiparasitic and pesticidal properties [8, 9]. Soursop fruit has been reported in folklore medicine for the prevention/management of type-2 diabetes and hypertension with little or no biochemical basis. This work was designed to investigate the effect of Soursop fruit parts (pericarp, pulp, and seed) extracts on key enzymes (*α*-amylase and *α*-glucosidase) linked with type-2 diabetes and [angiotensin-I converting enzyme (ACE)] hypertension. The distribution of phenolic contents and antioxidant properties of the Soursop fruit part extracts were subsequently assessed.

## 2. Materials and Methods

### 2.1. Sample Collection and Extraction

Soursop fruits were collected from botanical garden at the Federal University of Technology, Akure, Nigeria. Authentication of the fruit was carried out at the Department of Crop, Soil, and Pest Management, Federal University of Technology, Akure.

### 2.2. Sample Preparation

The fruits were sorted out and washed under running water to remove dirt. Thereafter, the fruits were separated into seeds, pulp, and fruit peels using table knife. They were chopped into small pieces by table knife, air-dried, and milled into a fine powder.

### 2.3. Preparation of the Extracts

The aqueous extracts were prepared by soaking 100 g of the powdered samples into 500 mL of distilled water for 24 h and filtered thereafter using Whatman filter paper. The filtrate was further spun in the centrifuge at 4000 ×g to obtain clear supernatants which were refrigerated and freeze-dried with the aid of freeze-drier. The dried powder was reconstituted in distilled water (1 : 100 w/v) and stored in the refrigerator for further analysis [10].

### 2.4. Reagents

Except otherwise stated, all chemicals used were of analytical grade. Glass distilled water was used.

### 2.5. *α*-Amylase Inhibition Assay

Appropriate dilutions of the extracts (500 *μ*L) in 0.02 M sodium phosphate buffer (pH 6.9 with 0.006 M NaCl) were added to 0.5 mg/mL of Hog pancreatic *α*-amylase (EC 3.2.1.1) and subsequently incubated at 25°C for 10 min. Then, 500 *μ*L of 1% starch solution in 0.02 M sodium phosphate buffer (pH 6.9 with 0.006 M NaCl) was added to each tube. The reaction mixture was incubated at 25°C for 10 min and stopped with 1.0 mL of dinitrosalicylic acid (DNSA) color reagent. Thereafter, the mixture was incubated in a boiling bath for 5 min and cooled to room temperature. The reaction mixture was further diluted with 10 mL of distilled water and the absorbance was read at 540 nm. The inhibitory effect of the extracts was calculated and expressed as percentage inhibition while acarbose was used as control [11].

### 2.6. *α*-Glucosidase Inhibition Assay

Fifty microliters of appropriate dilution of the extracts was added to 100 *μ*L of the *α*-glucosidase solution (1.0 U/mL) in 1.0 M phosphate buffer (pH 6.9) and incubated at 25°C for 10 min. Fifty microliters of 5 mM p-nitrophenyl-*α*-D-glucopyranoside solution in 0.1 M phosphate buffer (pH 6.9) was subsequently added. The reaction mixture was incubated at 25°C for 5 min and the absorbance was read at 405 nm in the spectrophotometer. The *α*-glucosidase inhibitory activity of the extract was calculated and expressed as percentage inhibition while acarbose was used as control [12].

### 2.7. Angiotensin-I Converting Enzyme (ACE) Inhibition Assay

Fifty microliters (50 *μ*L) of appropriate dilution of the extracts and ACE solution (50 *μ*L and 4 mU) was incubated at 37°C for 15 min. The enzymatic reaction was initiated by adding 150 *μ*L of 8.33 mM of the substrate Bz-Gly-His-Leu in 125 mM Tris-HCl buffer (pH 8.3) to the mixture. After incubation for 30 min at 37°C, the reaction mixture was arrested by adding 250 *μ*L of 1 M HCl. The Gly-His bond was then cleaved and the Bz-Gly produced by the reaction was extracted with 1.5 mL ethyl acetate. Thereafter the mixture was spun to separate the ethyl acetate layer. Then 1 mL of the ethyl acetate layer was transferred to a clean test tube and evaporated. The residue was redissolved in distilled water and its absorbance was measured at 228 nm. The ACE inhibitory activity was expressed as percentage inhibition while captopril was used as control [13].

### 2.8. Free Radical Scavenging Ability

The free radical scavenging ability of the extracts against DPPH (1,1-diphenyl-2-picrylhydrazyl) free radical was evaluated according to the method described by Gyamfi et al., 1999 [14]. Appropriate dilution of the extracts (1 mL) was mixed with 1 mL of 0.4 mM DPPH in methanolic solution. The mixture was left in the dark for 30 min and the absorbance was measured at 516 nm. The DPPH free radical scavenging ability was subsequently calculated.

### 2.9. Total Antioxidant Power

Total antioxidant power of the extracts was assessed using the ABTS radical model as described by Re et al., 1999 [15]. The ABTS radical was generated by reacting 7 mmol/L of ABTS aqueous solution with 2.45 mmol/L of K_2_S_2_O_8_ solution in the dark for 16 h and adjusting the Abs734 nm to 0.700 with ethanol. Two hundred microliters of the appropriate dilution of the sample extracts was added to 2.0 mL ABTS radical solution and the absorbance was measured at 734 nm after 15 min. The Trolox equivalent antioxidant capacity was subsequently calculated.

### 2.10. Hydroxyl (OH) Radical Scavenging Assay

The method of Halliwell and Gutteridge, 1981 [16], was used to determine the ability of the extracts to prevent Fe^2+^/H_2_O_2_ induced decomposition of deoxyribose. The extract 0–100 *μ*L was added to a reaction mixture containing 120 *μ*L of 20 mM deoxyribose, 400 *μ*L of 0.1 M phosphate buffer, and 40 *μ*L of 500 *μ*M FeSO_4_, and the volume was made up to 800 *μ*L with distilled water. The reaction mixture was incubated at 37°C for 30 min and the reaction was then stopped by the addition of 0.5 mL of 28% trichloroacetic acid. This was followed by addition of 0.4 mL of 0.6% thiobarbituric acid solution. The tubes were subsequently incubated in boiling water for 20 min. The absorbance was measured at 532 nm in a spectrophotometer and the OH scavenging ability was subsequently calculated.

### 2.11. Determination of Reducing Property

The reducing property of the extracts was determined by assessing the ability of the sample extract to reduce FeCl_3_ solution as described by Oyaizu, 1986 [17]. 2.5 mL aliquot was mixed with 2.5 mL of 200 mM sodium phosphate buffer (pH 6.6) and 2.5 mL of 1% potassium ferricyanide. The mixture was incubated at 50°C for 20 min. And then 2.5 mL of 10% trichloroacetic acid was added. This mixture was centrifuged at 650 rpm for 10 min. 5 mL of the supernatant was mixed with an equal volume of water and 1 mL of 0.1% ferric chloride. The absorbance was measured at 700 nm. The ferric reducing antioxidant property was subsequently calculated.

### 2.12. Fe^2+^ Chelation Assay

The Fe^2+^ chelating ability of the extracts was determined using the method of Minotti and Aust, 1987 [18], with a slight modification by Puntel et al., 2005 [19]. Freshly prepared 500 *μ*M FeSO_4_ (150 *μ*L) was added to a reaction mixture containing 168 *μ*L of 0.1 M Tris-HCl (pH 7.4), 218 *μ*L saline, and sample extracts (0–25 *μ*L). The reaction mixture was incubated for 5 min, before the addition of 13 *μ*L of 0.25% 1,10-phenanthroline (w/v). The absorbance was subsequently measured at 510 nm in a spectrophotometer. The Fe^2+^ chelating ability was subsequently calculated.

### 2.13. Determination of Total Phenol Content

The total phenol content was determined according to the method of Singleton et al., 1999 [20]. Briefly, appropriate dilutions of the extracts were oxidized with 2.5 mL of 10% Folin-Ciocalteu's reagent (v/v) and neutralized by 2.0 mL of 7.5% sodium carbonate. The reaction mixture was incubated for 40 min at 45°C and the absorbance was measured at 765 nm in the spectrophotometer. The total phenol content was subsequently calculated as gallic acid equivalent.

### 2.14. Determination of Total Flavonoid Content

The total flavonoid content was determined using a slightly modified method reported by Meda et al., 2005 [21]. Briefly 0.5 mL of appropriately diluted extracts was mixed with 0.5 mL of methanol, 50 *μ*L of 10% AlCl_3_, 50 *μ*L of 1 M potassium acetate, and 1.4 mL of water and allowed to incubate at room temperature for 30 min. The absorbance of the reaction mixture was subsequently measured at 415 nm; the total flavonoid content was subsequently calculated.

### 2.15. Data Analysis

The results of three (3) replicate experiments were pooled and expressed as mean ± standard deviation (SD). One-way analysis of variance (ANOVA) was used to analyse the mean and the post hoc treatment was performed using Duncan multiple test [22]. Significance was accepted at *P* ≤ 0.05. EC_50_ (extract concentration causing 50% antioxidant activity) was determined using nonlinear regression analysis with Graph Pad Prism version 5.00 for Windows.

## 3. Results

**Figure 1:**
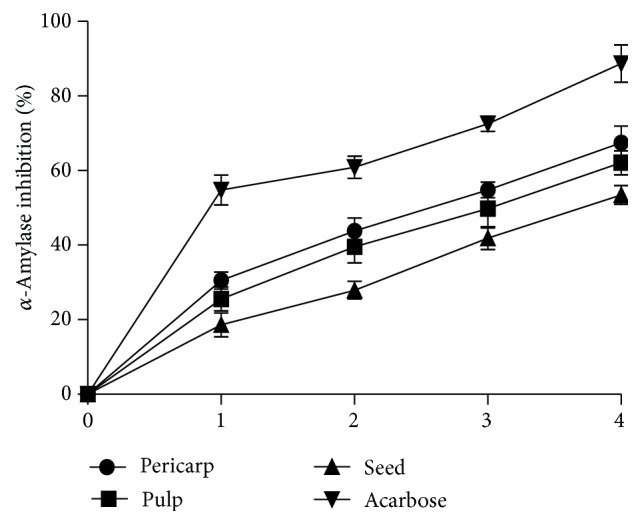
Percentage of *α*-glucosidase inhibition by extracts from Soursop fruit part and acarbose. The concentrations of the extract used for the plot are 0.00, 0.20, 0.40, 0.6, and 0.80 mg/mL. The concentrations of the acarbose used for the plot are 0.00, 10.00, 30.00, and 40.00 *μ*g/mL. Values represent mean of standard deviation of triplicate readings.

**Figure 2:**
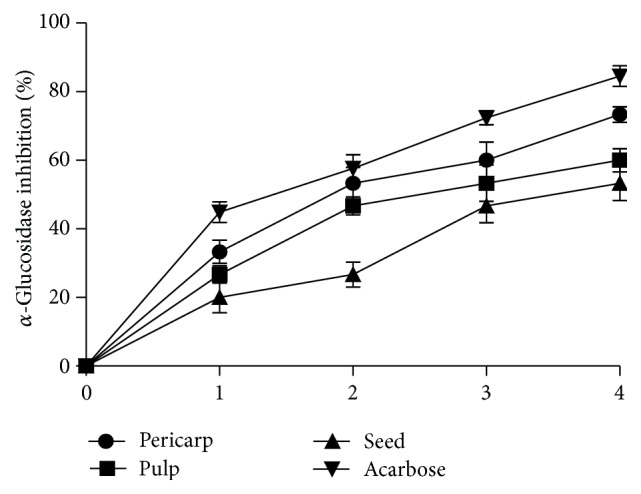
Percentage of *α*-glucosidase inhibition by extracts from Soursop fruit part and acarbose. The concentrations of the extract used for the plot are 0.00, 0.20, 0.40, 0.6, and 0.80 mg/mL. The concentrations of the acarbose used for the plot are 0.00, 10.00, 30.00, and 40.00 *μ*g/mL. Values represent mean of standard deviation of triplicate readings.

**Figure 3:**
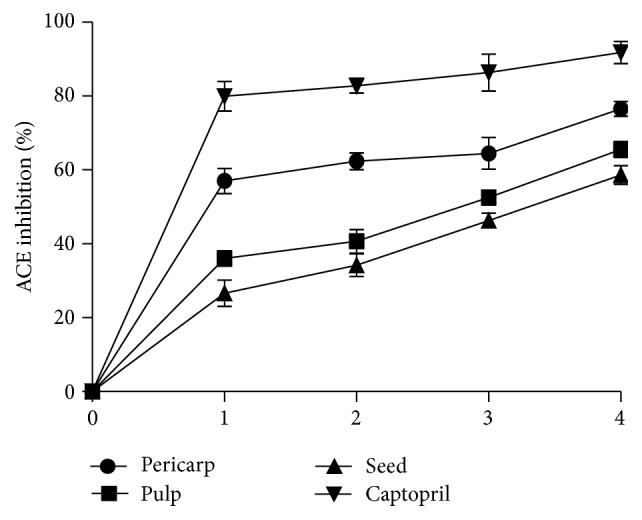
Angiotensin-I converting enzyme (ACE) inhibition by extracts from Soursop fruit part and captopril. The concentrations of the extract for the plot are 0.00, 0.05, 0.10, 0.20, and 0.25 mg/mL. The concentrations of captopril used for the plot are 0.00, 1.50, 2.50, 5.00, and 6.50 *μ*g/mL. Values represent mean of standard deviation of triplicate readings.

The* in vitro* *α*-amylase inhibitory effect of the aqueous extracts of different parts of Soursop fruit is presented in Figure 1. The result showed that all the extracts inhibited *α*-amylase activity in a concentration dependent manner (0–0.8 mg/mL). Nevertheless, the EC_50_ values (Table 1) showed that the pericarp extract (EC_50_ = 0.46 ± 0.03 mg/mL) had the highest inhibitory effect but lower inhibitory effect when compared to acarbose (IC_50_ = 9.51 ± 0.11 *μ*g/mL), while that of the seed (EC_50_ = 0.76 ± 0.03 mg/mL) had the least. Similarly, the* in vitro* *α*-glucosidase inhibitory effect of the acarbose and the extracts of different parts of Soursop fruit is presented in Figure 2. The result showed that all the extracts and acarbose inhibited *α*-glucosidase activity in a concentration dependent manner. Nevertheless, the pericarp extract (EC_50_ = 0.37 ± 0.03 mg/mL) had the highest inhibitory effect on *α*-glucosidase activity but lower inhibitory effect when compared to acarbose (12.97 *μ*g/mL) drug (Table 1). The* in vitro*  ACE inhibitory effect of the aqueous extracts of different parts of Soursop fruit is presented in Figure 3. The result showed that all the extracts inhibited ACE activity in a concentration dependent manner (0–0.25 mg/mL). However, the EC_50_ values (Table 1) showed that the pericarp extracts (EC_50_ = 0.03 ± 0.01 mg/mL) had the highest ACE inhibitory effect when compared to the pulp (EC_50_ = 0.14 ± 0.02 mg/mL) and seeds (EC_50_ = 0.20 ± 0.03 mg/mL) extracts. However, there was no significant (*P* > 0.05) difference between the pulp and the seed. Captopril had the highest ACE inhibitory effect compared to the entire sample extract with IC_50_ value of 0.13 *μ*g/mL.

**Figure 4:**
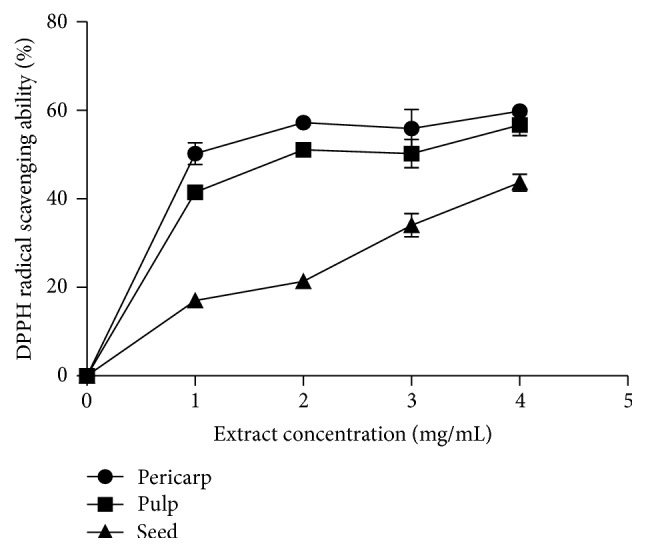
DPPH radical scavenging ability (%) of the aqueous extracts of the pericarp, pulp, and seed of Soursop fruit.

**Figure 5:**
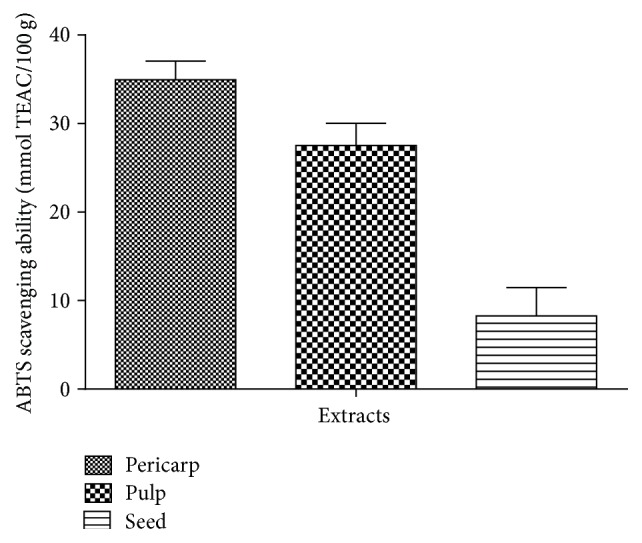
ABTS radical scavenging ability of the aqueous extracts of the pericarp, pulp, and seed of Soursop fruit.

**Figure 6:**
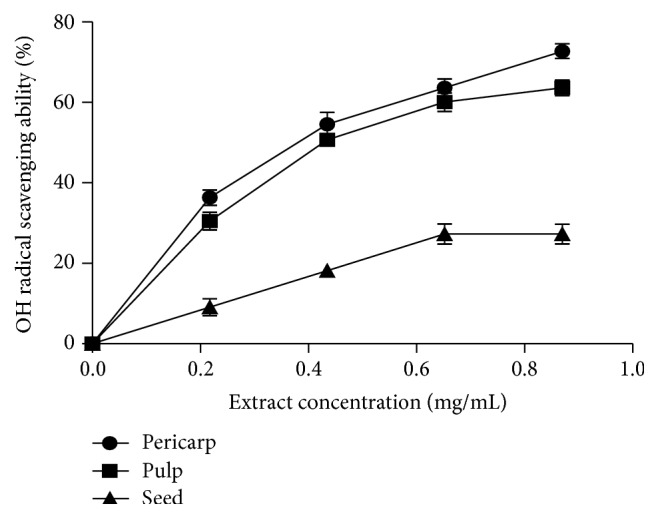
Hydroxyl (OH) radical scavenging ability (%) of the aqueous extracts of the pericarp, pulp, and seed of Soursop fruit.

**Figure 7:**
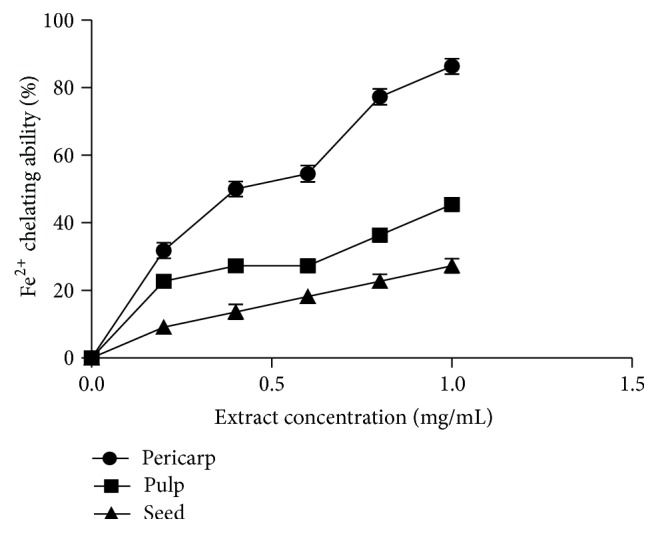
Fe^2+^ chelating ability (%) of the aqueous extracts of the pericarp, pulp, and seed of Soursop fruit.

Figure 4 showed the result of the DPPH free radical scavenging abilities of the extracts of Soursop fruit (pericarp, pulp, and seed). The results showed that all the extracts scavenged DPPH radical in concentration dependent manner (0–4.0 mg/mL). As shown in Table 1, extract of the pericarp had the highest DPPH radical scavenging ability (EC_50_ = 0.87 ± 0.01 mg/mL), when compared to the pulp (EC_50_ = 2.24 ± 0.07 mg/mL) and seed (EC_50_ = 5.44 ± 0.04 mg/mL) extracts.  Figure 5 revealed the result of the ABTS free radical scavenging abilities of the studied extracts. All the extracts scavenged ABTS free radical. The pericarp extract (34.9 ± 2.1 mmol TEAC/100 g) had the highest scavenging ability while the seed (8.3 ± 2.6 mmol TEAC/100 g) had the least. Furthermore, Figure 6 depicted the result of the hydroxyl (OH) radical scavenging ability of the extracts of Soursop fruit (pericarp, pulp, and seed). All the extracts significantly (*P* < 0.05) scavenged OH radical in concentration dependent manner (0–0.87 mg/mL). The extract of the pericarp had the highest OH radical scavenging ability (0.37 ± 0.01 mg/mL), while that of the seed had the least (2.25 ± 0.14 mg/mL).  Figure 7 showed the result of the Fe^2+^ chelating ability of the extracts of Soursop fruit part (pericarp, pulp, and seed). The extracts chelated Fe^2+^ in concentration dependent manner (0−1.0 mg/mL) and the pericarp extracts (EC_50_ = 0.39 ± 0.06 mg/mL) had the highest Fe^2+^ chelating ability, while the seed had the least (EC_50_ = 3.21 ± 0.05 mg/mL). The result of the ferric reducing antioxidant property of the Soursop fruit (pericarp, pulp, and seed) extract is presented in Table 2. The results showed that the reducing property of extracts ranged from 45.70 ± 4.28 mg AAE/100 g (seed) to 637.10 ± 9.11 mg AAE/100 g (pericarp).

Table 2 showed the total phenol and flavonoid contents of the extracts from the different parts of Soursop fruit understudied. The result showed that the total phenol content of the extract ranged from 50.51 ± 3.21 mg/100 g (seed) to 560.21 ± 6.22 mg/100 g (pericarp) while the total flavonoid contents ranged from 85.65 ± 7.63 mg/100 g (seed) to 275.45 ± 10.01 mg/100 g (pericarp). Overall, pericarp extract had the highest total phenol and flavonoid content followed by the pulp and the seed had the least.

## 4. Discussion

The observed inhibitory effect of the Soursop fruit part extracts on *α*-amylase and *α*-glucosidase activities* in vitro*  suggests the possible mechanism by which Soursop fruit parts exert their antidiabetic effect and may be part of the underlying basis for their folkloric use in the management/treatment of diabetes. Several studies have revealed that *α*-amylase and *α*-glucosidase activity have a great influence on blood glucose level and their inhibition could significantly reduce the postprandial increase of blood glucose [23]. It has also been established that reducing postprandial hyperglycemia is an important strategy towards type-2 management [24]. From this study, the pericarp of the fruit which had the highest total phenol and flavonoid content exhibited the highest *α*-amylase and *α*-glucosidase inhibitory effects. This is consistent with earlier studies where *α*-amylase and *α*-glucosidase inhibitory effect of plant foods are attributed to their phenolic constituents [25–27]. In addition, the fact that Soursop extracts significantly (*P* < 0.05) inhibited *α*-glucosidase more than *α*-amylase is of therapeutic importance at preventing the unpleasant side effects associated with strong synthetic *α*-amylase inhibitors, such as acarbose, and also agrees with previous studies that plant phenolic-rich extract inhibited *α*-glucosidase activity better than *α*-amylase activity [26, 28].

Although inhibitors of ACE activity are antihypertensive agents, they have been reported to reduce the risk of developing of type-2 diabetes [6, 28]. ACE inhibitors stimulate the release of bradykinin which, in turn, enhances the responsiveness of both muscle fiber and adipocytes to insulin utilization [6]. From the result, a strong correlation between the ACE inhibitory effects of the Soursop fruit extracts and the phenolic content was observed. The ability of the plant bioactive compounds such as phenolics could inhibit ACE activity [29–31]. It is proposed that phenolic phytochemicals showed a structure-function relationship in inhibiting ACE activity by chelating the active site zinc ion or inducing the formation of hydrogen bridges between the active site amino acid residues and the phenols [32]. This study showed strong correlation between the ACE inhibitory effect and the phenolic contents, as evidenced by the efficacy of the fruit pericarp with the highest phenolic contents. In line with increased interest in natural products as alternative to synthetic drugs, it is believed that these extracts would have little or no side effects when compared to these synthetic ACE inhibitor drugs such as captopril.

One of the risk factors in type-2 diabetes mellitus and its cardiovascular complication (hypertension) is oxidative stress. Oxidative stress has been reported to play a vital role in the etiology and development of type-2 diabetes and hypertension. Free radicals induced oxidative damage of pancreatic *β*-cells has been implicated in impaired insulin production/function, a major risk factor of diabetes development [33]. Also, oxidative damage to endothelia cell of the blood vessel could compromise the elasticity of the vessel resulting in hypertension or some other cardiovascular complications [34]. Thus combating oxidative stress could be a practical way to ensure holistic management of type-2 diabetes and hypertension. Therefore, it is imperative to investigate the antioxidant properties of Soursop fruit extracts, which have been reported in folklore medicine to be potent in the management of several diseases including diabetes and hypertension.

Polyphenolic compounds have shown antioxidant properties by reduction of Fe^3+^ to Fe^2+^, chelation of Fe, and mopping of radicals [26]. ABTS radical, a protonated radical, have characteristic absorbance maxima at 734 nm which decrease with the scavenging of the proton radicals [35], while the DPPH radical scavenging ability is through hydrogen ion donating ability [36]. This study showed that the extracts were able to scavenge (DPPH and ABTS) free radicals. The pericarp extract had the highest scavenging abilities as well as the highest total phenol and flavonoid contents.

Free radicals are capable of inducing oxidative damages in biomolecules via several reaction processes. One of such reactions is the Fenton reaction in which degradation of deoxyribose is initiated through Fe^2+^ catalyzed hydrogen peroxide (H_2_O_2_) decomposition to produce OH radicals [37]. The antioxidant properties of the Soursop fruit part extracts can also be measured by the ability to prevent degradation of deoxyribose via scavenging of hydroxyl (OH) radical and chelation of transition metals such as Fe^2+^. As observed in this study, OH radical scavenging and Fe^2+^ chelating abilities of the Soursop fruit parts (pericarp, pulp, and seed) extracts could be explored in the management oxidative stress-induced degenerative disease such as diabetes and hypertension. The report of this study is in line with previous studies indicating the ability of the phenolic compounds to chelate and/or deactivate transition metals and prevent such metals from participating in the initiation of lipid peroxidation and oxidative stress through metal catalysed reaction [4, 27, 38]. The pericarp from the fruit also exerted the highest Fe^2+^ chelating ability. This is further buttressed by the agreement between the antioxidant properties of the various Soursop fruit extracts and their phenolic contents.

Recently, polyphenolic compounds have become subjects of interest because of their beneficial effects on human health [26–28]. Numerous studies have shown that majority of the antioxidant activity of plants food is from phenolic compounds such as flavonoids, isoflavones, flavones, anthocyanins, catechin, and isocatechin rather than from vitamins C and E and *β*-carotene [27, 39, 40] and is believed to be due to their redox properties [41], which play a crucial role in adsorbing and neutralizing free radicals, quenching singlet and triplet oxygen, decomposing peroxides, chelating metal catalysts, and activating antioxidant enzymes [42]. According to Jiménez et al. [43], about sixteen phenolic compounds were reported to be predominantly present in Soursop fruit pulp. Cinnamic acid derivatives, p-coumaric acids together with several other minor compounds, were identified as the major phenolic compounds in Soursop fruit [43]. However, this study suggests that these phenolics may be well distributed in all the Soursop fruit part. Thus, this study showed that the pericarp had the highest total phenol content, followed by the pulp while the seed had the least phenolic contents (Table 2). This study further revealed that there are strong correlations between the phenolics contents and the biological activities studied. Therefore, phenolics (Table 2) in the Soursop fruit parts (pericarp, pulp, and seed) may be part of the active compound responsible for the antioxidant, antidiabetic, and antihypertensive and may provide a scientific basis of their use in folklore medicine. It is worth noting that the higher phenolic contents in the pericarp compared to the pulp and seed of the fruit could be due to the fact that the pericarp is more exposed to the environmental stress factors such as ultraviolet ray from the sunlight [44]. Stress factors provoke intense synthesis of phenolic compounds in the plant in order to forestall oxidative damage where the stress factors could confer to the plant cellular structures [45], unlike the pulp and seed that are protected by the edible portion of the fruit and therefore have less exposure to such stress factors. However, the values obtained for the extracts are lower than what was reported in some edible plant obtained in Iran and India [46] but higher than phenolics content in some selected tropical fruits from Malaysia [47]. To the best of our knowledge, this is the first time that the phenolic distribution and biological effects of Soursop fruit parts (pericarp, pulp, and seed) were reported.

## 5. Conclusion

This research investigated and revealed that Soursop fruit extracts possess antioxidant properties and were able to inhibit key enzymes relevant to type-2 diabetes mellitus (*α*-amylase and *α*-glucosidase) and hypertension (angiotensin-I converting enzyme)* in vitro*. The antidiabetic, antihypertensive, and antioxidant properties of the fruits part were strongly correlated to the phenolic contents. The combined enzyme inhibitory and antioxidant properties could be part of the biochemical rationale behind the traditional use of the Soursop fruit in the prevention and management of diabetes and hypertension. Nevertheless, this research has shown that Soursop's pericarp had the highest enzyme inhibitory and antioxidant properties compared to other parts (pulp and seed).

## Figures and Tables

**Table 1:** EC_50_ values for *α*-amylase, *α*-glucosidase, and ACE inhibitory properties and radicals (DPPH and OH) scavenging and Fe^2+^ chelating abilities of aqueous extracts of different parts of Soursop fruit (mg/mL) and standards (*μ*g/mL).

	Pericarp	Pulp	Seed	Acarbose	Captopril
*α*-Amylase	0.46 ± 0.03^a^	0.54 ± 0.03^b^	0.76 ± 0.03^c^	12.97 ± 0.11^a^	—
*α*-Glucosidase	0.37 ± 0.03^a^	0.51 ± 0.07^b^	0.73 ± 0.05^c^	9.51 ± 1.09^a^	—
ACE	0.03 ± 0.01^a^	0.14 ± 0.02^b^	0.20 ± 0.03^c^	—	0.13 ± 0.02^a^
DPPH radical	0.87 ± 0.01^a^	2.24 ± 0.07^b^	5.44 ± 0.04^c^	—	—
OH radical	0.37 ± 0.01^a^	0.46 ± 0.03^b^	2.25 ± 0.14^c^	—	—
Fe^2+^ chelation	0.39 ± 0.06^a^	1.78 ± 0.16^b^	3.21 ± 0.05^c^	—	—

Values represent means ± standard deviation of triplicate readings.

Values with the same superscript letter along the same row are not significantly different (*P* > 0.05).

**Table 2:** Total phenol and flavonoid contents and ferric reducing antioxidant property (FRAP) of aqueous extracts of different parts of Soursop fruit (mg/100 g).

	Pericarp	Pulp	Seed
Total phenol	560.21 ± 6.22^a^	430.29 ± 10.61^b^	50.51 ± 3.21^c^
Total flavonoid	275.45 ± 10.01^a^	100.01 ± 8.53^b^	85.65 ± 7.63^c^
FRAP	637.10 ± 9.11^a^	381.72 ± 8.22^b^	45.70 ± 4.28^c^

Values represent means ± standard deviation of triplicate readings.

Values with the same superscript letter along the same row are not significantly different (*P* > 0.05).
